# Management of acute pain in adults with sickle cell disease: the experience of the Clinical Hematology Department of the University of Dakar

**DOI:** 10.55730/1300-0144.5897

**Published:** 2024-07-17

**Authors:** Alioune Badara DIALLO, Moussa SECK, Mohamed KEITA, Sokhna Aissatou TOURE, Elimane Seydi BOUSSO, Baron NGASIA, Blaise Félix FAYE, Fatma DIENG, Saliou DIOP

**Affiliations:** Department of Hematology, Faculty of Medicine, Cheikh Anta Diop University, Dakar, Senegal

**Keywords:** Sickle cell disease, vaso-occlusive crisis, Senegal

## Abstract

**Background/aim:**

The evolution of sickle cell disease (SCD) is marked by the occurrence of painful episodes linked to the obstruction of microvessels by sickle cells, known as vaso-occlusive crisis (VOC). The aim of this work was to report the practical aspects of the management of acute pain in adults with SCD. Recommendations based on these practices are also provided.

**Materials and methods:**

This prospective, cross-sectional, descriptive, and analytical study was conducted over a four-month period of all sickle cell patients admitted to emergency departments for VOC. The parameters studied were sociodemographic, clinicobiological, therapeutic, and evolutionary.

**Results:**

There were 118 cases of VOC identified, representing a prevalence of 78.14% of sickle cell emergencies. The mean age of the patients was 28.41 years. The SS sickle cell phenotype accounted for 86.61% of the cases. Osteoarticular pain was the reason for admission for 88.39% of the patients; it was located in the lower limbs in 39.08% and in the spine in 27.1%. Pain intensity was moderate in 6.25% of the patients, intense in 31.25%, and unbearable in 55.55%. Multimodal analgesia was the most commonly used treatment method, combining those of levels one and two (74.31%) and levels one and three (8.25%). The mean dose of morphine administered was 17.14 mg when morphine alone was prescribed for titration, 13.57 mg when paracetamol and morphine were combined, and 15.83 mg when nefopam and morphine were combined. Clinical outcome was favorable in 68.87% of the cases.

**Conclusion:**

Wide variability was observed in the modalities of analgesic treatment of sickle cell VOC. These variations reflect different views on the appropriateness of opioids. This study highlights the efficacy of multimodal analgesia in the management of acute pain in patients with SCD, particularly in regard to morphine sparing. Context-specific recommendations will be needed to harmonize practices.

## 1. Introduction

Sickle cell disease (SCD) is a genetic disease characterized by the presence of an abnormal hemoglobin called hemoglobin S (HbS) in red blood cells. It is one of the most widespread genetic diseases in the world, mainly in sub-Saharan Africa, where the prevalence of the gene varies from 10% to 40% [1]. Vaso-occlusive crisis (VOC) is an acute painful complication of SCD related to the obstruction of microvessels by sickle red blood cells. Acute episodes of pain are the most common complication of SCD [2]. They can be managed at home; however, if they persist or if there are signs of severity, emergency hospital care is required. Despite numerous guidelines on the management of acute pain in patients with SCD, there is considerable variability in the way these painful episodes are managed by physicians [3].

In the sub-Saharan Africa setting, such observations reflect particularly different views on the availability of opioids and access to tertiary care centers. Misuse of opioids can lead to dependence, affecting patients’ quality of life.

The aim of this study was to report on the practical aspects of acute pain management in adults with SCD at the Clinical Hematology Department of the University of Dakar, with a view to develop recommendations based on these practices.

## 2. Materials and methods

This was a prospective, cross-sectional, descriptive, and analytical study conducted in the Hematological Emergency Department of the National Blood Transfusion Center in Dakar over a period of four months. All adult sickle cell patients admitted for VOC management who consented to participate in the study were included. The sociodemographic variables studied were age, sex, and profession. The clinical variables were disease-relative comorbidities, comorbid diseases other than SCD, sickle cell phenotype, and characteristics of the acute pain such as the duration, location, and intensity according to the numerical rating scale (NRS) [4]. Pain was classified as mild if the intensity was estimated at one to three on the NRS, moderate if the intensity was between four and five, intense if the intensity was between six and seven, and unbearable if the intensity was between eight and 10. Therapeutic aspects included the time taken for treatment and the drugs used in the emergency context. Analgesics were classified from levels one to three according to the World Health Organization analgesic ladder [5]. Evolutionary aspects were also studied in relation to the therapies used. A favorable outcome was concluded in cases of good clinical evolution with sedation of pain, normalization of vital signs, and clinical examination. The outcome was unfavorable if pain was not sedated or worsened after emergency treatment, or if the patient was deceased.

The data collected were analyzed using SPSS software. Quantitative variables were expressed as the mean ± standard deviation and median. Qualitative variables were expressed as percentages. The results were presented in the form of tables and graphs. Comparisons were made using the Student’s t-test for frequencies and chi-squared test for quantitative variables.

## 3. Results

### 3.1. General characteristics of patients

The median age of the patients was 26 (range: 16–70) years. The 20–40 age group was the most represented, at a rate of 77.6%. Females predominated with 62.7%, giving a sex ratio of 0.59. Eighty-two (69.6%) patients were unemployed. The most common professions among the sample were students, comprising 26.2%.

In this cohort, 88.4% of the patients had no significant medical history. Tuberculosis was observed in 4.2% of the patients. Sixteen (13.5%) patients had undergone cholecystectomy, and seven (5.9%) had undergone orthopedic surgery. Seventy (59.3%) patients had up-to-date vaccination status, and 12 (10.1%) had been vaccinated against COVID-19. Thirty-five (29.6%) patients had more than three VOCs per year. Thirty-three (28%) patients had a history of acute chest syndrome, and 15 (12.7%) had a history of priapism (Table 1).

There were 118 cases of VOC identified, representing a prevalence of 78.1% of sickle cell emergencies. The SS sickle cell phenotype accounted for 86.6% of the cases. The other phenotypes were represented by Sβ0 thalassemia (7.1%), SCD SC (3.5%), and Sβ+ thalassemia (2.6%). Based on hemoglobin electrophoresis, the mean HbS level was 86.4% for the SS phenotype and 74.65% for Sβ+thalassemia. The mean HbF level was 13% for the SS phenotype and 20% for Sβ0 thalassemia (Table 2).

### 3.2. Clinical data

The median time between the onset of symptoms and a visit to the emergency department was 2.85 days, with a range of one to 10 days. The triggering factor of VOC was found in 97% of the patients, including infection in 37.5%, intense physical activity in 36.93%, stress in 15%, and dehydration 3.4%.

Osteoarticular pain was the reason for admission in 88.39% of the patients. It was located in the lower limbs in 39.08% and in the spine in 27.1%. The other sites were the upper limbs (25.7%) and ribs (8.9%) (Figure). Pain intensity was moderate in 6.25% of the patients, intense in 31.25%, and unbearable in 55.55%. The mean pain intensity at NRS for the SS sickle cell patients was 7.03 (±1.06). It was 6.62 (±1.06), 6.25 (±2.21), and 6.00 (±1.00), respectively, for sickle cell phenotypes Sβ0 thalassemia, SC, and Sβ+thalassemia (Table 1).

### 3.3. Therapeutic aspects

Prehospital management at home was observed in 84.7% of the patients, primarily involving the use of oral analgesics: level 1 (paracetamol, nefopam) alone (24.2%), or a combination of levels 1 and level 2 (codeine, tramadol) (75.7%).

All the patients received emergency pain management. Analgesic treatment consisted of a combination of levels 1 and 2 analgesics prescribed for 74.31% of the patients, a combination of levels one and three for 8.25%, level two alone for 7.33%, and level three alone for 6.42%. The combination of nefopam and tramadol was prescribed for 30.68% of the patients for pain of moderate intensity at 6.30 (±1.64) on the NRS. The combination of paracetamol and morphine was prescribed for 26.48% of the patients for pain of moderate intensity at 8.28 (±1.11). Morphine alone was prescribed for 6.48% of the patients for pain of average intensity of 9.51 (±0.78) (Table 2). Morphine was prescribed for 42.22% of the patients. The mean dose of morphine administered was 17.14 mg when morphine alone was prescribed for titration. This dose was 13.57 mg when paracetamol and morphine were combined and 15.83 mg when nefopam and morphine were combined. Parenteral hydration with 0.9% isotonic saline was prescribed for 82.78% of the patients.

### 3.4. Evolutionary aspects

Clinical outcome was favorable in 68.87% of the patients. The average length of stay in the emergency department was 3.47 h, with extremes ranging from 1 to 6 h; this varied according to the intensity of the pain and the analgesics used (Table 3). Eighteen patients with persistent VOC were admitted for continuous hospitalization, representing 15.25% of the patients.

## 4. Discussion

VOC, or sickle cell crisis, is a common painful complication of SCD. Acute episodes of severe pain are the primary reason that these patients seek medical care in hospital emergency departments.

Osteoarticular pain was the main reason for admission in the current series (88.39%), with a predominant location in the lower limbs (39.08%) and spine (27.1%). This is in line with the literature data [6–8]. This osteoarticular pain was very intense according to the numerical scale in most cases (55.5%). The different beta-globin haplotypes can be correlated with pain intensity, with relatively less painful crisis for the Arab-Indian and Senegalese haplotypes [9]. However, the subjectivity of the numerical scale may constitute a bias in the assessment of pain in sickle cell patients seen in emergency departments. In addition, a comparison between patients who made little use of the emergency department (less than three visits per year) and those who made frequent use of this care system showed that the latter had a more serious disease, which caused more intense pain [10]. In the current series, 33.3% of the patients had more than three VOCs per year. In addition, pain intensity was significantly higher in homozygous sickle cell patients (Table 2). This could be explained by the relatively higher hemoglobin S level and the relatively lower fetal hemoglobin level in homozygous sickle cell patients compared with other sickle cell phenotypes.

In the present study, hydration and analgesics were the most commonly used treatments in emergency departments. Parenteral hydration was exclusively with isotonic saline. The recommendations of a group of African experts on the management of SCD recommend a solution composed as follows: 500 mL of 5% glucose serum with 2 g of sodium chloride and 0.75 g of potassium chloride or Ringer’s lactate or 0.9% isotonic saline; with an infusion quantity of 2 L/m^2^/24 h in adults [11]. Hydration, by whatever route, corrects the dehydration of sickle cells, and therefore their vaso-occlusive and adhesion effects [3,12].

Multimodal analgesia was the most commonly used modality in the current study. Analgesic treatment depended on the intensity of pain according to the NRS. Overall, moderate pain was treated by combining level one and two analgesics, and unbearable pain was treated by combining level one and three analgesics or with a level three analgesic alone. Multimodal analgesia was effective in the current series, given the shorter stay in emergency when analgesics were combined. International recommendations for effective multimodal analgesia favor the prescription of opioid analgesics [13,14]. The choice of a level one analgesic in combination with morphine depended essentially on the prescriber (paracetamol, morphine, metamizole), with a preference for paracetamol. The efficacy of this combination was established in various recommendations [13,14]. Although nefopam is now widely prescribed for the management of VOC, there are no studies to date on the efficacy of this analgesic in this situation [15]. There were no cases of nonsteroidal antiinflammatory drugs (NSAIDs) being prescribed during the current study. This therapeutic class has not been shown to be effective in sickle cell crisis, despite its use in combination with morphine. A study carried out by a French reference center showed that ketoprofen reduced neither the intensity of pain nor the duration of the crisis [16]. It should also be noted that the use of NSAIDs in an infectious context should be avoided because of the risk of serious infectious complications. The mean dose of morphine administered was lower when combined with a level one analgesic (paracetamol, nefopam) compared with morphine alone. These results underline the value of multimodal analgesia, particularly in morphine sparing, which considerably reduces the dose of morphine administered and therefore its side effects. The opioid analgesic intervention for pain management in SCD during an acute painful crisis is effective, but improper management can lead to dependence [17]. Furthermore, the risk of acute chest syndrome has been significantly associated with high systemic exposure to morphine. Morphine may facilitate respiratory deterioration by eliciting a decrease in oxygen saturation, by inducing histamine release, or through an as-yet-unidentified mechanism [18].

Pain management in VOC is complex and requires rapid and effective care. There is great variability in the way in which painful episodes are managed, particularly in sub-Saharan African setting. Variations in practice reflect different views on the appropriateness of opioids, the efficacy of parenteral administration and the risk of opioid dependence. This study highlights the effectiveness of multimodal analgesia in the management of acute pain in patients with SCD, particularly in regard to morphine sparing. Context-specific recommendations will be needed to harmonize practices in order to avoid or minimize morphine-dependence among this patient population.

## Figures and Tables

**Figure:**
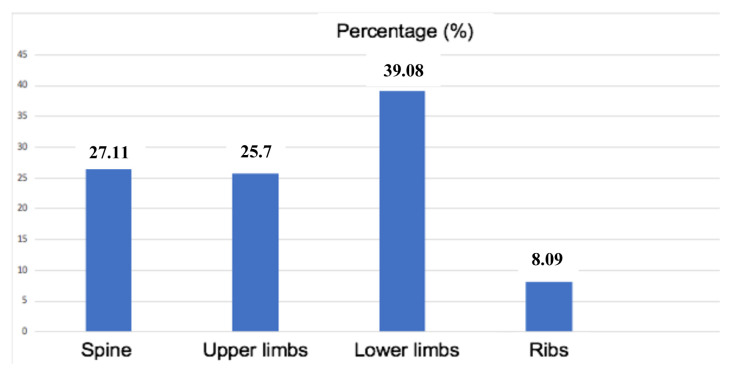
Location of osteoarticular pain.

**Table 1:** General characteristics of patients.

Characteristics	Values	Percentages (%)

Median age (years) (range)	26 (16–70)	

Sex		
- Males (M)	44	37.2
- Females (F)	74	62.7
- Ratio (M/F)	0.59	

Professions		
- Unemployed	82	69.6
- Students	31	26.2
- Others	5	4.2

History		
- >3 VOC per year	35	29.6
- Priapism	15	12.7
- Acute chest syndrome	33	28
- Up-to-date vaccination status	70	59.3
- Tuberculosis	5	4.2
- Cholecystectomy	16	13.5
- Orthopedic surgery	7	5.9

**Table 2:** Biological characteristics of the different electrophoretic profiles (values in %) and intensity of pain according to the numerical rating scale (NRS). SD* Standard deviation.

Electrophoretic profiles (%)	A	A2	S	C	F	NRS of pain (Mean ± SD)
Sβ+ thalassemia	-	1.97 ± 0.38	65.43 ± 3.55	-	27.00 ±10.00	6.00 ± 1.00
Sβ0 thalassemia	-	3.20 ± 0.11	74.65 ± 7.60	-	20.00 ± 7.00	6.62 ± 1.06
SC	-	-	52.00 ± 5.80	42.65 ± 0.20	2.30 ± 0.44	6.25 ± 2.21
SS	-	3.47 ± 1.54	86.45 ± 7.52	-	13.00 ± 8.00	7.03 ± 1.06

**Table 3:** Distribution of different analgesic approaches, their corresponding pain levels and the length of stay at the emergency department. SD* Standard deviation.

Analgesics	Numerical scale Mean ± standard deviation	Percentage (%)	Length of stay Mean ± SD (Hours)
Metamizole, Tramadol	7.00 ± 1.00	3.70	3,98 ± 0.88
Morphine	9.51 ± 0.78	6.48	1.45 ± 0.01
Nefopam, morphine	8.66 ± 1.15	2.78	1.33 ± 0.57
Paracetamol, morphine	8.28 ± 1.11	26.48	2.25 ± 0.50
Paracetamol, Tramadol, Morphine	9.40 ± 0.90	4.63	3.5 ± 0.93
Tramadol	6.75 ± 0.95	2.14	
Tramadol, Morphine	7.50 ± 1.50	1.85	1.30 ± 0.10
Paracetamol, Tramadol	6.30 ± 1.50	21.26	4.00 ± 0.93
Nefopam, Tramadol	6.30 ± 1.64	30.68	3.86 ± 0.60

## References

[1] Modell B Global epidemiology of haemoglobin disorders and derived service indicators Bulletin of the World Health Organization 2008 6 480 487 10.2471/BLT.06.036673 PMC2647473 18568278

[2] Brandow AM DeBaun MR Key Components of Pain Management for Children and Adults with Sickle Cell Disease Hematology/Oncology Clinics of North America 32 3 535 550 10.1016/j.hoc.2018.01.014 PMC6800257 29729787

[3] Jacob E Pain management in sickle cell disease Pain Management Nursing 2 4 121 131 10.1053/jpmn.2001.26297 11748547

[4] Breivik H Borchgrevink PC Allen SM Rosseland LA Romundstad L Assessment of pain British Journal of Anaesthesia 101 1 17 24 10.1093/bja/aen103 18487245

[5] Anekar AA Hendrix JM Cascella M 2024 WHO Analgesic Ladder StatPearls StatPearls Publishing http://www.ncbi.nlm.nih.gov/books/NBK554435/ 32119322

[6] Kato GJ Piel FB Reid CD Gaston MH Ohene-Frempong K Sickle cell disease Nature Reviews Disease Primers 4 1 18010 10.1038/nrdp.2018.10 29542687

[7] Dodo R Zohoun A Baglo T Mehou J Anani L Urgences drépanocytaires au Service des Maladies du Sang du Centre National Hospitalier Universitaire-Hubert Koutoukou Maga de Cotonou, Benin Pan African Medical Journal 30 10.11604/pamj.2018.30.192.15931 (in French) PMC6235509 30455821

[8] Platt OS Thorington BD Brambilla DJ Milner PF Rosse WF Pain in Sickle Cell Disease: Rates and Risk Factors New England Journal of Medicine 325 1 11 16 10.1056/NEJM199107043250103 1710777

[9] Brousse V Colin Y Pereira C Arnaud C Odièvre MH Erythroid Adhesion Molecules in Sickle Cell Anaemia Infants: Insights Into Early Pathophysiology EBioMedicine 2 2 154 157 10.1016/j.ebiom.2014.12.006 PMC4485482 26137540

[10] Mbika Cardorelle A Okoko A Mouko A Les crises vaso-occlusives de l’enfant drépanocytaire à Brazzaville Archives de Pédiatrie 17 3 295 296 10.1016/j.arcped.2009.11.009 (in French) 20034770

[11] Guide de prise en charge de la drépanocytose en Afrique 2018 64 65 (in French)

[12] Okomo U Meremikwu MM Fluid replacement therapy for acute episodes of pain in people with sickle cell disease The Cochrane Collaboration Cochrane Database of Systematic Reviews (CD005406-pub4) John Wiley and Sons, Ltd 10.1002/14651858.CD005406.pub4 17443589

[13] Brandow AM Carroll CP Creary S Edwards-Elliott R Glassberg J American Society of Hematology 2020 guidelines for sickle cell disease: Management of acute and chronic pain Blood Advances 4 12 2656 2701 10.1182/bloodadvances.2020001851 PMC7322963 32559294

[14] Recommandations professionnelles Prise en charge de la drépanocytose chez l’enfant et l’adolescent Haute Autorité de la Santé (HAS) France 2010 (in French)

[15] Okpala I Tawil A Management of Pain in sickle-Cell Disease Journal of the Royal Society of Medicine 95 9 456 458 10.1177/014107680209500909 PMC1279994 12205212

[16] Bartolucci P El Murr T Roudot-Thoraval F Habibi A Santin A A randomized, controlled clinical trial of ketoprofen for sickle-cell disease vaso-occlusive crises in adults Blood 114 18 3742 3747 10.1182/blood-2009-06-227330 19717646

[17] Feliu MH Wellington C Crawford RD Wood M Edwards L Opioid Management and Dependency Among Adult Patients with Sickle Cell Disease *Hemoglobin* 35 5–6 485 494 10.3109/03630269.2011.610914 21910605

[18] Kopecky EA Jacobson S Joshi P Koren G 2004 Systemic Exposure to Morphine and the Risk of Acute Chest Syndrome in Sickle Cell Disease Clinical Pharmacology and Therapeutics 75 3 140 146 10.1016/j.clpt.2003.10.007 15001964

